# The G2A Receptor Controls Polarization of Macrophage by Determining Their Localization Within the Inflamed Tissue

**DOI:** 10.3389/fimmu.2018.02261

**Published:** 2018-10-01

**Authors:** Katharina Kern, Stephan M. G. Schäfer, Jennifer Cohnen, Sandra Pierre, Tabea Osthues, Neda Tarighi, Stefan Hohmann, Nerea Ferreiros, Bernhard Brüne, Andreas Weigert, Gerd Geisslinger, Marco Sisignano, Klaus Scholich

**Affiliations:** ^1^Institute of Clinical Pharmacology, Pharmazentrum Frankfurt, University Hospital Frankfurt, Frankfurt, Germany; ^2^Project Group Translational Medicine and Pharmacology, Fraunhofer Institute for Molecular Biology and Applied Ecology IME, Frankfurt, Germany; ^3^Faculty of Medicine, Institute of Biochemistry I, Goethe-University Frankfurt, Frankfurt, Germany

**Keywords:** G2A, GPCR, macrophage, polarization, migration, acute inflammation, pain

## Abstract

Macrophages are highly versatile cells, which acquire, depending on their microenvironment, pro- (M1-like), or antiinflammatory (M2-like) phenotypes. Here, we studied the role of the G-protein coupled receptor G2A (GPR132), in chemotactic migration and polarization of macrophages, using the zymosan-model of acute inflammation. G2A-deficient mice showed a reduced zymosan-induced thermal hyperalgesia, which was reversed after macrophage depletion. Fittingly, the number of M1-like macrophages was reduced in the inflamed tissue in G2A-deficient mice. However, G2A activation was not sufficient to promote M1-polarization in bone marrow-derived macrophages. While the number of monocyte-derived macrophages in the inflamed paw was not altered, G2A-deficient mice had less macrophages in the direct vicinity of the origin of inflammation, an area marked by the presence of zymosan, neutrophil accumulation and proinflammatory cytokines. Fittingly neutrophil efferocytosis was decreased in G2A-deficient mice and several lipids, which are released by neutrophils and promote G2A-mediated chemotaxis, were increased in the inflamed tissue. Taken together, G2A is necessary to position macrophages in the proinflammatory microenvironment surrounding the center of inflammation. In absence of G2A the macrophages are localized in an antiinflammatory microenvironment and macrophage polarization is shifted toward M2-like macrophages.

## Introduction

Macrophages are heterogeneous immune cells that exhibit a high plasticity and can be roughly classified by two polarized end states named M1 (proinflammatory) and M2 (antiinflammatory) (1). Monocyte-derived macrophages differentiate from Ly6C^hi^ blood monocytes and infiltrate tissues during inflammation. Once having crossed the endothelium and entered the inflamed tissue they differentiate to macrophages and polarize toward M1 or M2 phenotypes depending on their microenvironment (2–4). During the different phases of inflammation, macrophage subsets exhibit various phenotypic states within this M1/M2 spectrum whereby changes in the microenvironment induce reversible transcriptional reprogramming (1, 5). M1-like macrophages, which were polarized by stimuli such as interferon γ (IFNγ) or toll-like receptor (TLR)-ligands, exhibit strong antimicrobial capacities by producing high levels of proinflammatory mediators, whereas M2-like macrophages participate in clearance of cell debris and efferocytosis during resolution (1, 6).

Apoptotic cells release so-called “find-me” signals to attract phagocytes and ensure their engulfment. In macrophages the G-protein coupled receptor G2A (GPR132) is essential for efferocytosis of apoptotic neutrophils (7–9). Lysophosphatidylcholines (LPCs) of different chain length promote chemotaxis toward apoptotic cells through G2A (10–12) although LPCs are not direct G2A agonists. Instead LPCs prevent spontaneous internalization of G2A, promote redirection of internalized G2A and its redistribution to the cell membrane as well as supporting G2A dimerization (13, 14).

G2A is predominantly expressed in immune cells, including macrophages, (9, 15) where it mediates efferocytosis of apoptotic neutrophils (7, 8, 16). G2A has originally been described as a proton sensor, exhibits a low sensitivity to pH fluctuations (17) and serves as a promiscuous receptor for oxidized free fatty acids such as 9-hydroxyoctadecadienoic acid (9-HODE) (18, 19) and some hydroxyeicosatetraenoic acids (HETEs) (19). Recently, lactate was also shown to activate G2A in macrophages and to induce polarization toward M2-like phenotypes (20). G2A-deficient mice are through young adulthood indistinguishable from wild type mice. However, in lymphocytes G2A plays an important homeostatic role by controlling peripheral lymphocyte numbers by regulating the threshold of TCR-dependent activation and proliferation. With increasing age G2A^−/−^ mice develop secondary lymphoid organ enlargement associated with increased T- and B-cell numbers, causing at an age of more than 1 year to a slowly progressive wasting syndrome, which is associated with lymphocytic infiltration into various tissues (21).

We studied the role of G2A in polarization of macrophages during an acute zymosan-induced inflammation, a common model for toll-like receptor-2-mediated inflammation, which mediates immune responses against a wide array of infectious agents including gram-positive bacteria, virus, protozoa, and fungi (22). While the overall number of infiltrating macrophages was not significantly altered, G2A-deficient mice had a reduced number of macrophages in the proinflammatory microenvironment in the direct vicinity of the origin of inflammation. As consequence, the macrophages are localized in absence of G2A in an antiinflammatory microenvironment and less M1-like macrophages are found in the inflamed tissue. Thus, although G2A activation has been demonstrated to promote M2-like phenotypes in a tumor environment (20), during an acute inflammation G2A promotes indirectly M1-like phenotypes by positioning the macrophages in an inflammatory microenvironment.

## Experimental procedures

### Animals

We used 8–12 weeks old C57BL/6N mice (Janvier, Le Genest-Saint-Isle, FR) and G2A-deficient (G2A^−/−^) mice (Jackson laboratories, Bar Harbor, ME, USA). All experiments were performed according to the recommendations of the Guide of the Care and Use of Laboratory Animals of the National Institutes of Health and were approved by the local ethics committee (Approval numbers FK1093, FK1066, FK1029, FK1046, and FK1061).

### Antibodies

Antibodies used were against 5-lipoxygenase (Cayman Chemicals, catalog number 160402), autotaxin (Phoenix Pharmaceuticals, catalog number H-008-29), CD45 (Miltenyi Biotech, clone 30F11.1 catalog number 130-091-609), CD80 (Biolegend, clone 16-10A1 catalog number 104706), CD86 (Biolegend, clone GL-1 catalog number 105007), CD206 BioRad, clone MR5D3 catalog number MCA2235GA, and Biolegend, clone C068C2 catalog number 141707), F4-80 (Biolegend clone BM8 catalog number 123107), IL1β (eBiosciences, clone NJTEN3 catalog number) 11-7114-82, Ly6C (eBiosciences, clone HK1.4 catalog number 17-5932-80), Ly6G (BioLegend, clone 1A8 catalog number 127613), and TNFα (Miltenyi Biotech, clone REA636 catalog number 130-109-720).

### Zymosan-induced thermal hyperalgesia

In all cases the experimenter was unaware of the treatments or the genotypes of the mice. 10 μl Zymosan (3 mg/ml in PBS, catalog number Z4250, Sigma-Aldrich, Darmstadt, Germany) were administered subcutaneously into the plantar side of one hind paw. Thermal thresholds were determined using the Hargreaves test (23). Briefly, mice were put on a heated glass plate (32°C) for 1–2 h to allow accommodation. For measuring thermal pain thresholds, the midplantar area was stimulated with a flashlight until fast withdrawal of the paw occurred. Depletion of macrophages with clodronate followed published protocols. Briefly, clodronate-containing and PBS-containing liposomes were purchased from clodronateliposomes.org (Vrije University, Netherlands). To ablate macrophages, mice were injected i.p. on day -4 and day -1 with 200 μl of suspension with a total of 1 mg encapsulated clodronate (24, 25). Control mice were injected i.p. with 200 μl PBS Liposomes, respectively.

### FACS analyses

FACS analyses for inflamed paws and bone marrow-derived macrophages were performed as follows (26): The tissues were cut in pieces and incubated for 45 min at 37°C in 500 μl of lysis buffer [DMEM without FCS, 3 mg/ml Collagenase IA, 1 U/ml DNAse I (Promega, Mannheim, Germany)]. Tissue lysis was stopped by addition of 500 μl lysis-stop buffer (DMEM, 10% FCS) and the suspension was passed through a 70 μm filter. The cells were centrifuged at 1300 × g for 5 min, resuspended in erythrocyte lysis buffer (135 mM NH4Cl, 10 mM NaHCO3, 0.1 mM Na-EDTA, pH 7.2) and incubated for 4 min at room temperature. After centrifugation (1300 × g, 5 min) the cells were washed with HBSS buffer without Ca2+ and Mg2+, 10% citrate, and resuspended in 150 μl of FACS buffer (PBS, 1% FCS, 0.1% NaN3). The cells were stained with fluorescence-labeled antibodies in FACS buffer for 30 min at 4°C. Afterwards cells were washed twice with FACS buffer, resuspended in 200 μl FACS/PFA buffer (FACS buffer, 1% buffered formaldehyde) and kept at 4°C until FACS measurement. All FACS analyses were performed on a FACSCanto flow cytometer (Becton Dickinson, Heidelberg, Germany).

For FACS sorting cells were prepared as described above with the exception that NaN3 was omitted from the buffers. The cells were stained with antibodies against F4-80, Ly6C, and either CD86 or CD206 in 50 μl PBS/1% FCS in for 30 min at 4°C. Then the cells were washed once with PBS and sorted using FACS Aria III (BD Biosciences) (27, 28).

### Bone marrow-derived macrophages

For generation of bone marrow-derived macropghages (28) femur and tibia of hind legs from adult mice were taken and the ends of the bones were cut. Bone marrow was extracted by centrifugation with 10,000 × g for 10 s. The cells were differentiated in RPMI1640 with L-glutamine (Life Technologies), 10% FCS, 100 U/ml penicillin and 100 μg/ml streptomycin and 20 ng/ml mCSF (catalog number AF-315-02, Peprotech, Hamburg, Germany) for 7 days. The macrophages were stimulated with LPS (100 ng/ml, catalog number L4391, Sigma Aldrich, Darmstadt, Germany) and/or (±)9-HODE (1 μM, catalog number 38400, Cayman Chemical, Ann Arbor, MI, USA) for 24 h before analysis. Cytokines were determined by ELISA (R&D Systems, Wiesbaden, Germany) following the manufacturer's instructions. Griess test was performed by mixing supernatant and Griess reagent (Merck Millipore, Darmstadt, Germany) at a ratio of 1:1 and measuring absorbance at 540 nm 20 min later.

### Migration assay

Bone marrow-derived macrophages were serum starved overnight and then stained with 10 μM 5(6)-CFDA, SE (catalog number. 51014, Biotium, Fremont, CA, US) for 10 min. They were detached with accutase, washed and transferred into 5 μM polycarbonate membrane transwell inserts. Inserts were transferred to plates supplied with RPMI medium and incubated for 2 h in absence or presence of 10% serum or 9-HODE (1 μM). Migrated macrophages were lysed and fluorescence intensities were measured using an Infinite F220 (TECAN, Mainz, Germany).

### Real-time RT-PCR

Bone marrow-derived macrophages were differentiated for 7 days using 20 ng/ml M-CSF. Monocytes were isolated from peripheral blood of naïve mice with F4-80 magnetic beads (MACS Miltenyi, Bergisch-Gladbach, Germany). Macrophages were isolated from inflamed paws 24 h after zymosan injection by FACS sorting. RNA was isolated using mirVana miRNA Isolation Kit (Ambion, Darmstadt, Germany) and transcribed using First Strand cDNA Synthese Kit (Thermo Fisher Scientific Darmstadt, Germany). RT-PCR was performed for G2A (Mm02620285_s1) and GAPDH (Mm99999915_g1) with a 7500 Fast System and TaqMan Gene Expression Assay (Thermo Fisher Scientific, Darmstadt, Germany). Relative mRNA expression is shown as 2^−ΔΔ*CT*^.

### Multi-epitope-ligand cartography (MELC)

The MELC technology is an immunhistological imaging method that uses directly labeled antibodies to allow the sequential visualization of 20–40 proteins on the same sample (28, 29). Briefly, tissues were embedded in tissue freezing medium (Leica Microsystems, Nussloch, Germany), cryosections of 10 μm thickness were applied on silane-coated coverslips, fixed in 4% paraformaldehyde in PBS for 15 min, permeabilized with 0.1% Triton X100 in PBS for 15 min and blocked with 3% BSA in PBS for 1 h. The sample was placed on the stage of a Leica DM IRE2. By a robotic process, the tissue sample was incubated for 15 min with a fluorescence-labeled antibody and then washed with PBS. Afterwards, the phase contrast and fluorescence signals were imaged by a cooled charge-coupled device camera (Apogee KX4; Apogee Instruments, Roseville, CA). To delete the fluorescence signal, a bleaching step was performed. Then a post-bleaching image was recorded and the next antibody was applied. After all antibodies were imaged fluorescence images produced by each antibody were aligned pixel-wise using the corresponding phase contrast images. Then the images were corrected for illumination faults using flat-field correction and the post-bleaching images were subtracted from the respective following fluorescence images for complete elimination of remaining fluorescence.

### Analysis of MELC images

Images were analyzed for coexpression using an established protocol (28, 29). To determine coexpression of proteins the relative immunofluorescence intensities for all the antibodies were determined in a single pixel using the TIC Experiment Viewer software (Meltec, Magdeburg, Germany). The minimum intensity (value 0) was set individually for each antibody to eliminate background staining. Maximum intensity (value 1) was set for each antibody using the brightest pixel in its image. A protein was defined as present in an individual pixel when its signal reached at least 10 percent of the maximal signal strength. For every cell the protein expression was compared in 3–4 pixels to ensure consistent expression levels. To calculate the percentage of F4-80^+^/Ly6C^+^ and F4-80^+^/Ly6C^−^ macrophages, first all cells positive for F4-80 were counted in the respective areas (for example 0–100 μm distance from the border of the zymosan covered area). Only cells outside of blood vessels, which could be clearly distinguished from neighboring cells were included in the analysis to avoid false positive or negative signals. Then coexpression of F4-80^+^ and Ly6C^+^ were determined as described above. The number of Ly6C-positive and -negative F4-80-expressing cells was finally calculated as percent of all F4-80-expressing cells present in a defined region.

Expression profiles were generated using the Image J function Plot Profiles. Three linear expression profiles were taken for each image. The distance was calculated for each pixel in regard to the border of the zymosan containing area (0 μm).

### Cytokine measurements

Cytokines within the paw edemas were determined 24 h after zymosan injection using ELISA kits from R&D Systems. The edemas were isolated, homogenized and lysed for 30 min in Cell Lysis Buffer 2 (R&D Systems) before analyses. To normalize cytokine concentrations to the protein content, protein concentrations were determined using the Bradford protein assay.

### Lactate measurements

Tissue from inflamed paws was prepared at the indicated times and lysed in PBS by sonication. Lactate was measured using the Promega Lactate-Glo Assay (Mannheim, Germany) following the manufacturer's instructions.

### Lipid measurements

LC-MS/MS analysis of LPAs, LPCs, HETEs, and HODEs were deermined using a hybrid triple quadrupole-ion trap mass spectrometer QTrap 5500 (Sciex, Darmstadt, Germany), an Agilent 1260 HPLC binary pump, column oven, and degasser (Waldbronn, Germany), and a HTC Pal autosampler (Zwingen, Switzerland). For details please see Supplemental Experimental Procedures.

### Data analysis and statistics

All data are presented as mean±S.E.M. To determine statistically significant differences Two-way or One-way variants of ANOVA or Student's *t*-test were applied. *p* < 0.05 were considered statistically significant.

## Results

### Decreased zymosan-induced hyperalgesia in G2A^−/−^ mice is macrophage-dependent

**Figure 1:**
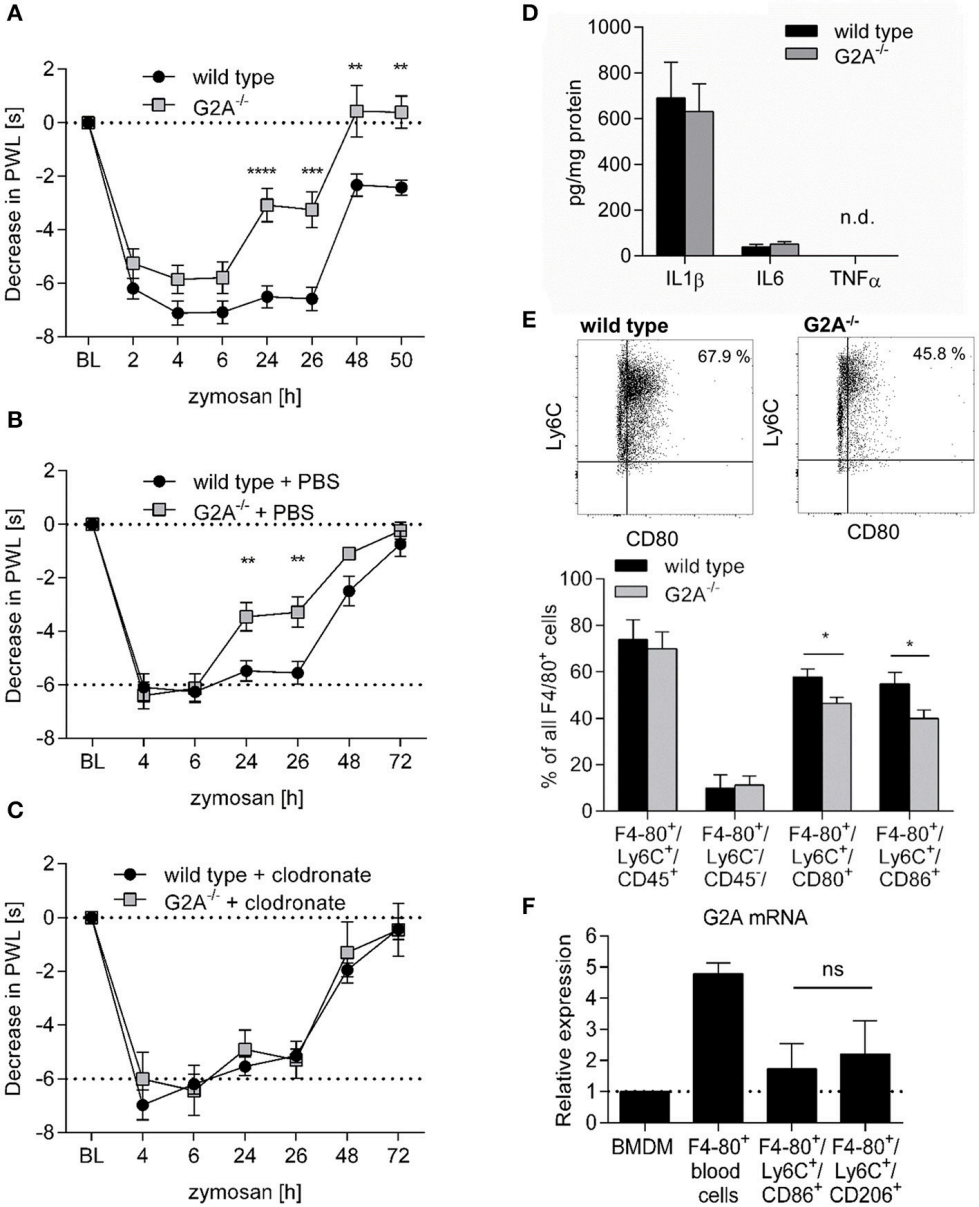
Decreased zymosan-induced hyperalgesia in G2A^−/−^ mice is macrophage-dependent. **(A)** Zymosan-induced thermal paw withdrawal latencies (PWL) were determined in wildtype (WT) and G2A^−/−^ mice at the indicated time points after injection of 10 μl zymosan (3 mg/ml). Data are shown as mean ±S.E.M. (*n* = 6). Two-Way ANOVA with Bonferroni *post-hoc* test ^**^p < 0.001, ^***^p < 0.0001, ^****^p < 0.00001 as compared to WT. **(B,C)** Zymosan-induced thermal PWLs after macrophage depletion. Mice were treated with PBS-containing liposomes **(B)** or clodronate-containing liposomes **(C)** 1 and 4 days prior the zymosan injection (10 μl, 3 mg/ml). Data are shown as mean ± S.E.M. (*n* = 6). Two-Way ANOVA with Bonferroni *post-hoc* test. ^**^p < 0.001 as compared to WT. **(D)** IL1β, IL6, and TNFα levels in paws of wild type and G2A^−/−^ mice 24 h after zymosan injection (10 μl, 3 mg/ml) were determined by ELISA. Data are shown as mean ± S.E.M. (*n* = 5). **(E)** FACS analysis of macrophage subsets in inflamed paws of wild type and G2A^−/−^ mice 24 h after zymosan-injection (10 μl, 3 mg/ml). Data are shown as mean ± S.E.M. (*n* = 4–8). Unpaired two-tailed Student‘s *t*-test. ^*^p < 0.05. **(F)** RT-PCR analysis for G2A expression in bone marrow-derived macrophages (BMDM), monocytes and macrophages. Bone marrow-derived macrophages differentiated for 7 days using 20 ng/ml M-CSF, monocytes were isolated from peripheral blood of naïve mice and macrophages were isolated from inflamed paws 24 h after zymosan-injection (10 μl, 3 mg/ml). Data are shown as mean ± S.E.M. (*n* = 3). Unpaired two-tailed Student‘s *t*-test, n.s.: not significant.

Toll-like receptor (TLR)-mediated paw inflammation is a common model to study mechanisms of acute tissue inflammation and inflammatory pain (nociception). Here, we compared the nociceptive response of wild type and G2A-deficient mice during paw inflammation induced by the TLR2-agonist zymosan. We observed a significantly decreased thermal hyperalgesia in G2A^−/−^ mice 24 h after zymosan-injection (Figure 1A), a time point where the inflamed tissue is characterized by a strong infiltration of monocyte-derived macrophages (26, 28). Since G2A is expressed in several myeloid cells, besides macrophages, we treated wild type and G2A-deficient mice with PBS- (Figure 1B), or clodronate-containing liposomes (Figure 1C) to deplete selectively phagocytes (30). While PBS-containing liposomes had no effect on the nociceptive response, clodronate-induced phagocyte depletion reversed the reduced hyperalgesia in G2A^−/−^ mice, but had no effect on thermal hyperalgesia in wild type mice (Figure 1C). FACS analysis of the inflamed paws showed that the clodronate-treatment decreased the number of monocyte-derived macrophages in the inflamed paw 24 h after zymosan injection while the number of CD11c^+^/CD11b^+^-positive dendritic cells was not altered (Figure S1) suggesting that macrophages mediate the antinociceptive effect in G2A-deficent mice. The level of typical proinflammatory cytokines (IL1β, IL6, and TNFα) did not differ between wild type and G2A-deficient mice 24 h after zymosan injection (Figure 1D) and FACS analysis of inflamed paws of wild type and G2A^−/−^ mice, showed similar macrophage numbers for both resident (F4-80^+^/Ly6C^−^/CD45^−^) and monocyte-derived macrophages (F4-80^+^/Ly6C^+^/CD45^+^) (Figure 1E; Figure S2A). However, G2A^−/−^ mice had significantly reduced numbers of proinflammatory monocyte-derived macrophages (F4-80^+^/Ly6C^+^/CD86^+^ and F4-80^+^/Ly6C^+^/CD80^+^) (Figure 1E; Figure S2B). Notably, this was not due to a differential expression of G2A in the different macrophage subpopulations, since G2A mRNA levels were similar in M1-like (F4-80^+^/Ly6C^+^/CD86^+^) and M2-like (F4-80^+^/Ly6C^+^/CD206^+^) macrophages isolated from edemas 24 h after zymosan-injection (Figure 1F). G2A mRNA was also detected in bone marrow derived macrophages and F4-80^+^ cells isolated from murine blood (Figure 1F). Thus, so far the data show that the antinociceptive effect in G2A-deficient mice is mediated by macrophages and that the number of M1-macrophages is decreased in G2A^−/−^ mice.

### G2A-activation does not alter M1-polarization in bone marrow-derived macrophages

**Figure 2:**
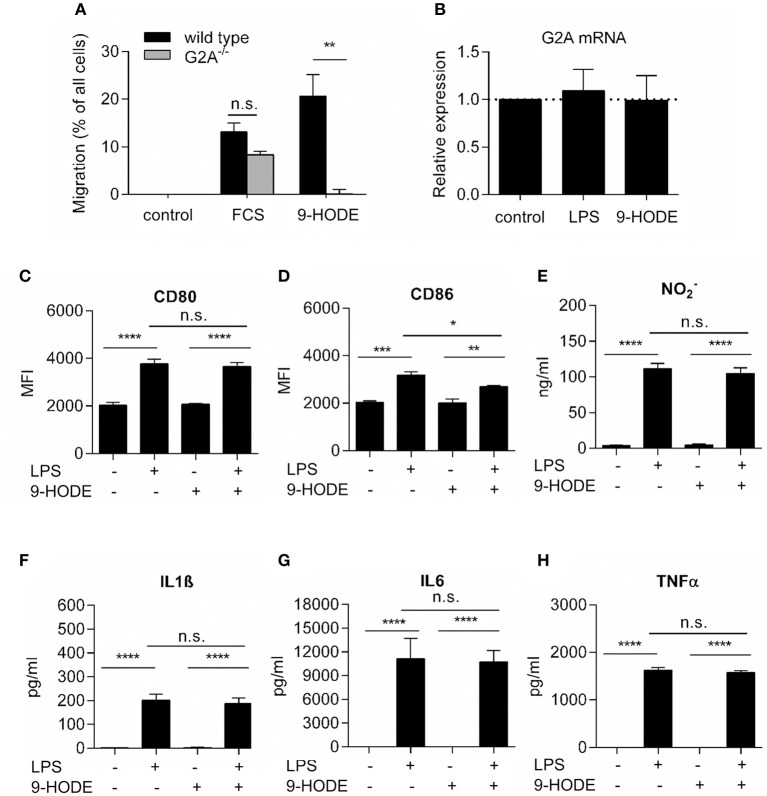
G2A-activation does not alter proinflammatory markers in bone marrow-derived macrophages. **(A)** G2A-mediated macrophage migration. Data are shown as mean ± S.E.M. (*n* = 3–4). Migration of BMDMs from wild type and G2A^−/−^ mice was not induced (control) or induced by serum and 9-HODE. Unpaired two-tailed Student‘s *t*-test. ^**^p < 0.01 as compared to control. **(B)** RT-PCR analysis for G2A expression in BMDM, differentiated for 7 days with 20 ng/ml M-CSF and stimulated with 100 ng/ml LPS or 1 μM 9-HODE for 24 h. Data are shown as 2^(−ΔΔ*CT*)^, mean ± S.E.M. (*n* = 4). **(C–H)** Marker expression by bone marrow-derived macrophages from wild type mice after stimulation with 100 ng/ml LPS or 1 μM 9-HODE for 24 h. CD80 and CD86 expression were analyzed by FACS, nitrite using the Griess test and IL1β, IL6, and TNFα by ELISA. Data are shown as mean ± S.E.M. (*n* = 4–5). One-Way ANOVA with Bonferroni *post-hoc* Test ^*^p < 0.05, ^**^p < 0.001, ^***^p < 0.0001, ^****^p < 0.00001.

Since G2A^−/−^ mice had decreased numbers of M1-like macrophages, we studied whether or not G2A has a direct effect on macrophage polarization toward M1-like phenotypes. To ensure that G2A expression is sufficient to mediate biological activity, we first tested the G2A-agonist 9-HODE for its known ability of G2A to induce chemotaxis in macrophages (10). Indeed, 9-HODE induced migration in wild type but not in G2A-deficient bone marrow-derived macrophages (Figure 2A). Also, G2A mRNA levels in bone marrow-derived macrophages from wild type mice were not altered after treatment with 9-HODE and/or with the proinflammatory stimulus lipopolysaccharides (LPS) (Figure 2B). In regard to the expression of markers for proinflammatory macrophages, the positive control LPS increased in wild type cells the expression of CD80 and CD86 as well as the release of nitrite or the proinflammatory cytokines Interleukin (IL)-1β, IL-6, and tumor necrosis factor α (TNFα) (Figures 2B–H). However, activation of G2A by 9-HODE in absence or presence of LPS had no effect on the expression levels of these markers (Figures 2B–H). Likewise, 9-HODE had in G2A-deficient macrophages no effect on the expression level of these markers (Figures S3A–F). In summary, G2A activation does not seem to directly regulate macrophage polarization toward M1-phenotypes, which suggests an indirect mechanism causing the reduction of M1-like macrophages in G2A-deficient mice.

### Loss of G2A decreases the number of monocyte-derived macrophages at the site of inflammation and reduces neutrophil efferocytosis

**Figure 3:**
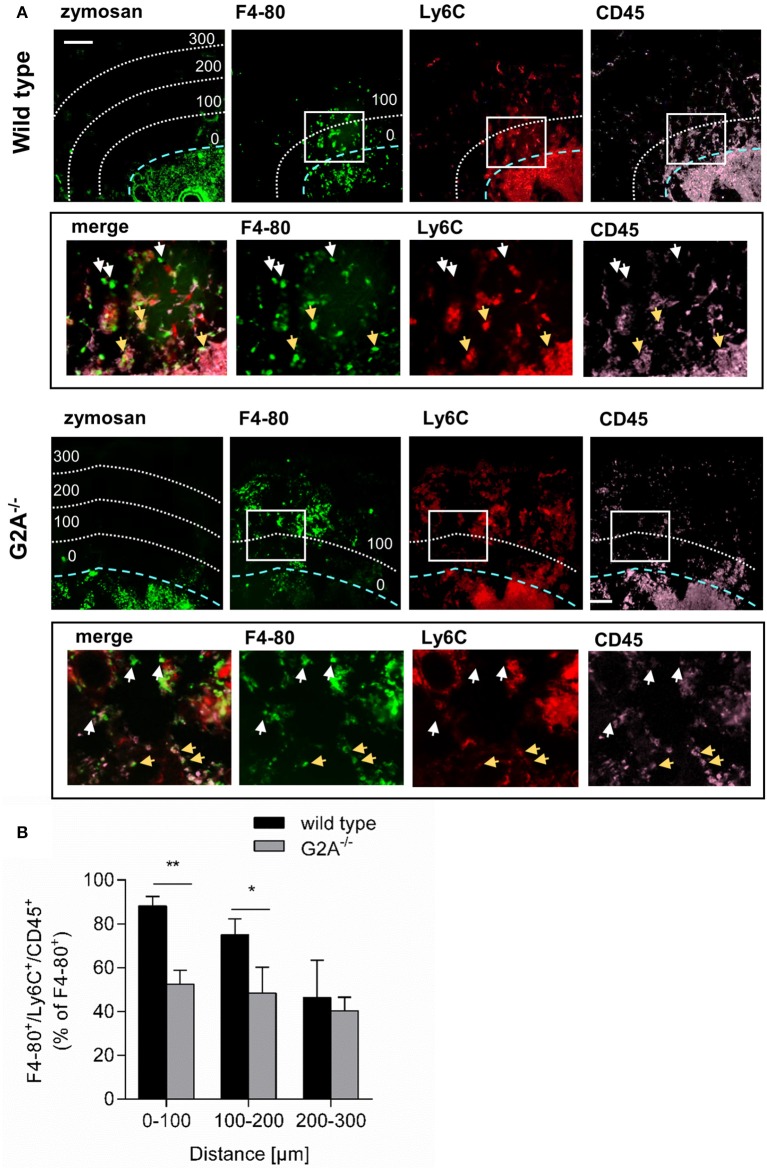
G2A-deficieny decreases the number of monocyte-derived macrophages at the center of inflammation. **(A,B)** Inflamed paws from wild type and G2A^−/−^ mice were prepared 24 h after zymosan injection (10 μl, 3 mg/ml) for immunohistological analysis of CD45, Ly6C, and F4–80. Representative MELC images **(A)** and quantitative analysis **(B)** of monocyte-derived macrophage distribution are shown. The blue dotted line shows the border of the zymosan-containing area and the white dotted lines indicate 100, 200, and 300 μm distance from the border. All images are shown in false colors. White squares depict the area shown in the lower panels (black box). White arrows depict F4-80^+^/Ly6C^+^/CD45^+^ monocyte-derived macrophages and light brown arrows depict F4-80^+^/Ly6C^−^/CD45^−^ resident macrophages. Data are shown as mean ± S.E.M. (*n* = 3–4). Unpaired one-tailed Student‘s *t*-test. ^*^p < 0.05, ^**^p < 0.01. The white bar indicates 100 μm.

Previously it has been demonstrated that G2A mediates the migration of macrophages toward apoptotic cells (11). We hypothesized that in absence of G2A the migration of macrophages toward the origin of inflammation is reduced and that therefore less macrophages acquire a proinflammatory phenotype. To test this hypothesis, we injected FITC-labeled zymosan into hind paws of wild type and G2A^−/−^ mice to be able to localize the center of inflammation. Using the MELC system for sequential multiple immunohistology, we determined the number of monocyte-derived macrophages (F4-80^+^/Ly6C^+^/CD45^+^) in relation to the distance from the border of the zymosan-covered region. Here, only cells outside of blood vessels, which could be clearly distinguished from neighboring cells were included in the analysis. We found that in wild type mice the percentage of F4-80^+^/Ly6C^+^/CD45^+^ macrophages of all F4-80^+^ cells dropped with increasing distance from the zymosan-containing area from 88% (0–100 μm) to around 50% (200-300 μm) (Figures 3A,B). In contrast, in G2A^−/−^ mice the percentage of F4-80^+^/Ly6C^+^/CD45^+^ macrophages was consistently around 50% of all macrophages (Figure 3B). This percentage was in the direct vicinity of the injected zymosan (0–200 μm) significantly lower than in wild type mice.

**Figure 4:**
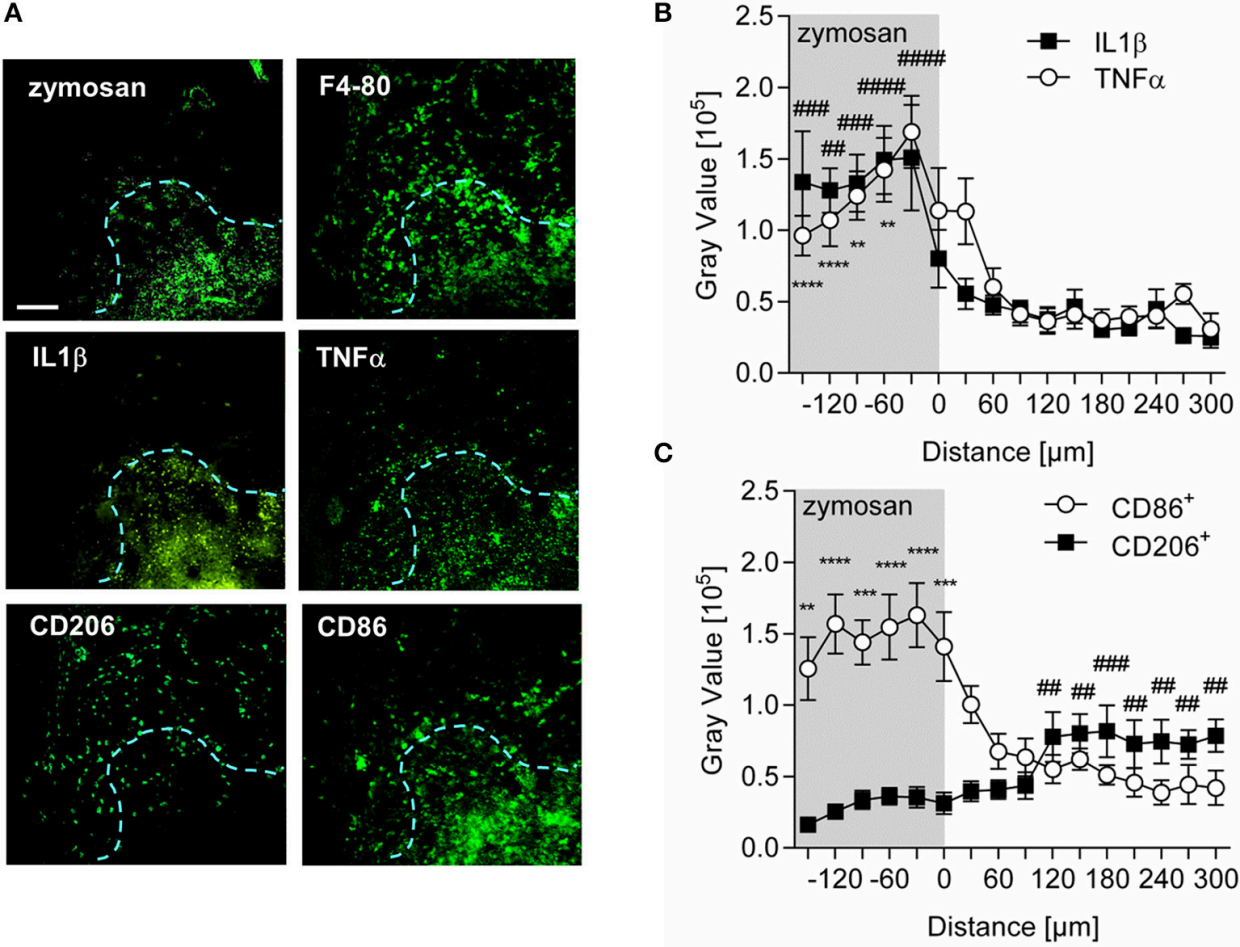
M1-like macrophages are located in a proinflammatory microenviroment around the center of inflammation. **(A)** Representative immunohistological images of a MELC analysis for proinflammatoty cytokines and macrophage markers in paws 24 h after zymosan-injection (10 μl, 3 mg/ml). The blue dotted line shows the border of the zymosan-containing area. The white bar indicates 100 μm. **(B,C)** Distribution of TNFα and IL1β **(B)** or CD86^+^ and CD206^+^ **(C)** in inflamed paws 24 h after zymosan injection. Images were analyzed using pixel intensities (gray values) generated by linear plot profiles. Data are shown as mean ± S.E.M. (*n* = 7–10). One-way-ANOVA with Bonferroni *post-hoc* test. ^**^,##*p* < 0.001, ^***^,###*p* < 0.0009, ^****^, ####*p* < 0.0001. IL1β, TNFα, and CD86^+^ compared to value at 300 μM. CD206^+^ compared to value at −150 μm.

Analysis of the expression patterns of the prototypical proinflammatory mediators TNFα and IL1β in wild type mice showed that both cytokines were localized to the center of inflammation and their presence decreased dramatically with increasing distance to the zymosan-containing area (Figures 4A,B). A similar analysis of expression profiles for M1-like (CD86) macrophages, localized CD86^+^ expressing cells primarily within the area labeled by the injected zymosan (Figures 4A,C). In contrast, CD206^+^ expressing (M2-like) cells were preferentially located outside the zymosan-containing area in distances of >100 μm from the zymosan border (Figures 4A,C). Thus, the localization of proinflammatory monocyte-derived macrophages correlates with a proinflammatory microenvironment as marked by the presence of proinflammatory cytokines.

**Figure 5:**
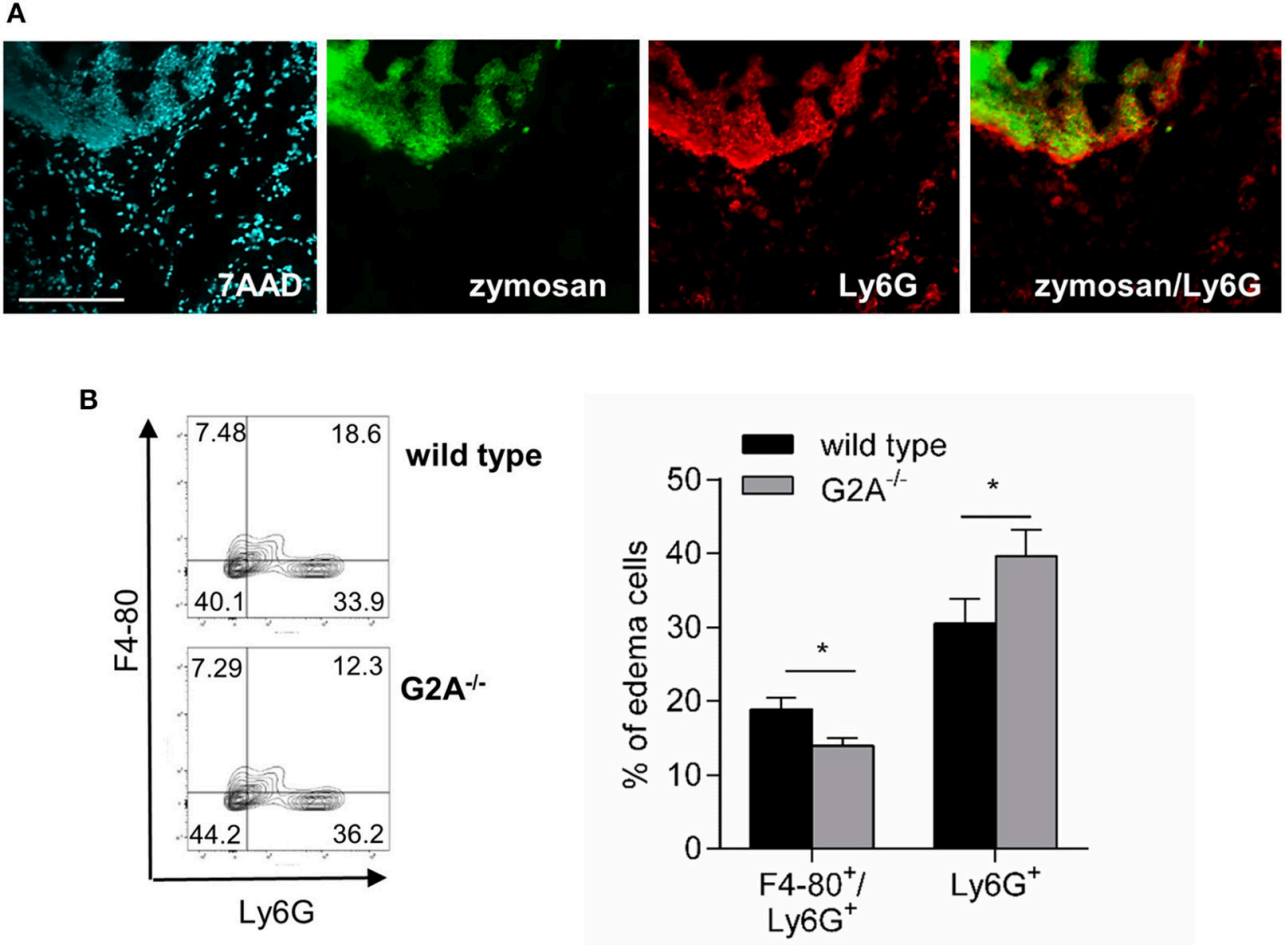
Loss of G2A reduces neutrophil efferocytosis. **(A)** Neutrophils (Ly6G) are localized at the zymosan-covered area. Representative MELC staining of paws 24 h after zymosan-injection (10 μl, 3 mg/ml). 7AAD was used as nuclear stain. The white bar indicates 100 μm. **(B)** FACS analysis of macrophages/neutrophil complexes (F4-80/ Ly6G) and neutrophils (Ly6G) in the inflamed tissue 24 h after zymosan-injection (10 μl, 3 mg/ml). Data are shown as mean ± S.E.M. (*n* = 8). Unpaired one-tailed Student‘s *t*-test. ^*^p < 0.05.

Apoptotic neutrophils release “find-me” signals, which can be detected by G2A, and induce macrophage migration and subsequently clearance of neutrophils by macrophages (7, 8). In the zymosan-model neutrophils were localized in wild type mice in and around the zymosan-containing area (Figure 5A) and therefore in the region, where the number of monocyte-derived macrophages is decreased. Fittingly we found in the inflamed paws of G2A-deficient mice elevated neutrophil numbers (Figure 5B) and decreased numbers of F4-80^+^/Ly6G^+^ double-positive cells, which define macrophage/neutrophil complexes and are indicative for macrophages clearing apoptotic neutrophils (Figure 5B).

**Figure 6:**
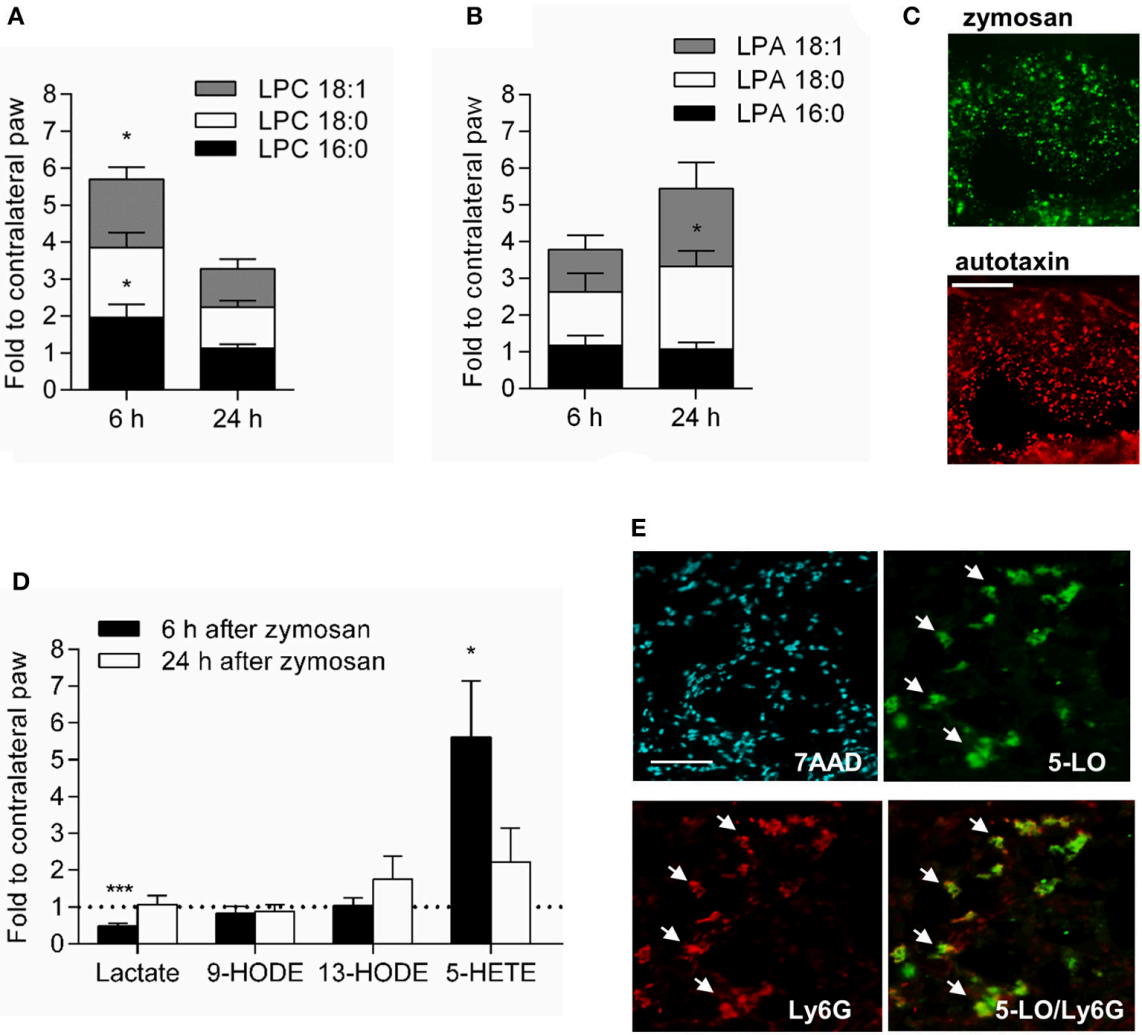
Lipids, which mediate G2A-dependent migration, are increased during zymosan-induced inflammation. **(A,B)** Concentration of LPCs **(A)** and LPAs **(B)** 6 and 24 h after injection of zymosan (10 μl, 3 mg/ml). Data are shown as mean ± S.E.M. (*n* = 5). Unpaired two-tailed Student‘s *t*-test. ^*^p < 0.05 as compared to the contralateral values. **(C)** Representative immunohistological images showing autotaxin staining in the zymosan covered region 24 h after zymosan injection (10 μl, 3 mg/ml). Scale bar 100 μm. **(D)** Concentration of some G2A agonists in the inflamed tissue 6 and 24 h after zymosan-injection (10 μl, 3 mg/ml). Data are normalized for concentrations determined in untreated paws and are shown as mean ± S.E.M. (*n* = 5). Unpaired two-tailed Student‘s *t*-test. ^*^p < 0.05. **(E)** Neutrophils (Ly6G) express 5-lipoxygenase (5-LO). Representative immunohistological staining of paws 24 h after zymosan-injection (10 μl, 3 mg/ml). The white bar indicates 100 μm. Arrows depict Ly6G^+^/5-LO^+^-cells. ^***^p < 0.0001.

Apoptotic neutrophils release LPCs, in particular LPC 16:0, 18:0, and 18:1, which induce a directed G2A-mediated macrophage migration (10, 11). The combined levels of these three LPCs were significantly increased 6 h after zymosan-injection in wild type mice (Figure 6A). 24 h after zymosan injection the LPC levels decreased, whereas the levels of some of their metabolites (i.e., lysophosphatidic acid (LPA) 18:0 and 18:1) increased (Figure 6B). Fittingly autotaxin, the enzyme, which converts LPCs to LPAs, was localized in the zymosan-containing area (Figure 6C). Most other known G2A agonists, such as lactate, 9-HODE or 13-HODE, were not significantly increased in the inflamed paw (Figure 6D). However, zymosan-injection increased temporarily the levels for the G2A-agonist 5-HETE (19) (Figure 6D) and 5-lipoxygenase, the enzyme responsible for 5-HETE synthesis was expressed in neutrophils at the site of inflammation (Figure 6E). However, given that the observed effects on macrophages are occurring in a relative small clearly defined region, we would like to point out that other G2A agonists might as well be locally increased but are not detected in this analysis.

## Discussion

**Figure 7:**
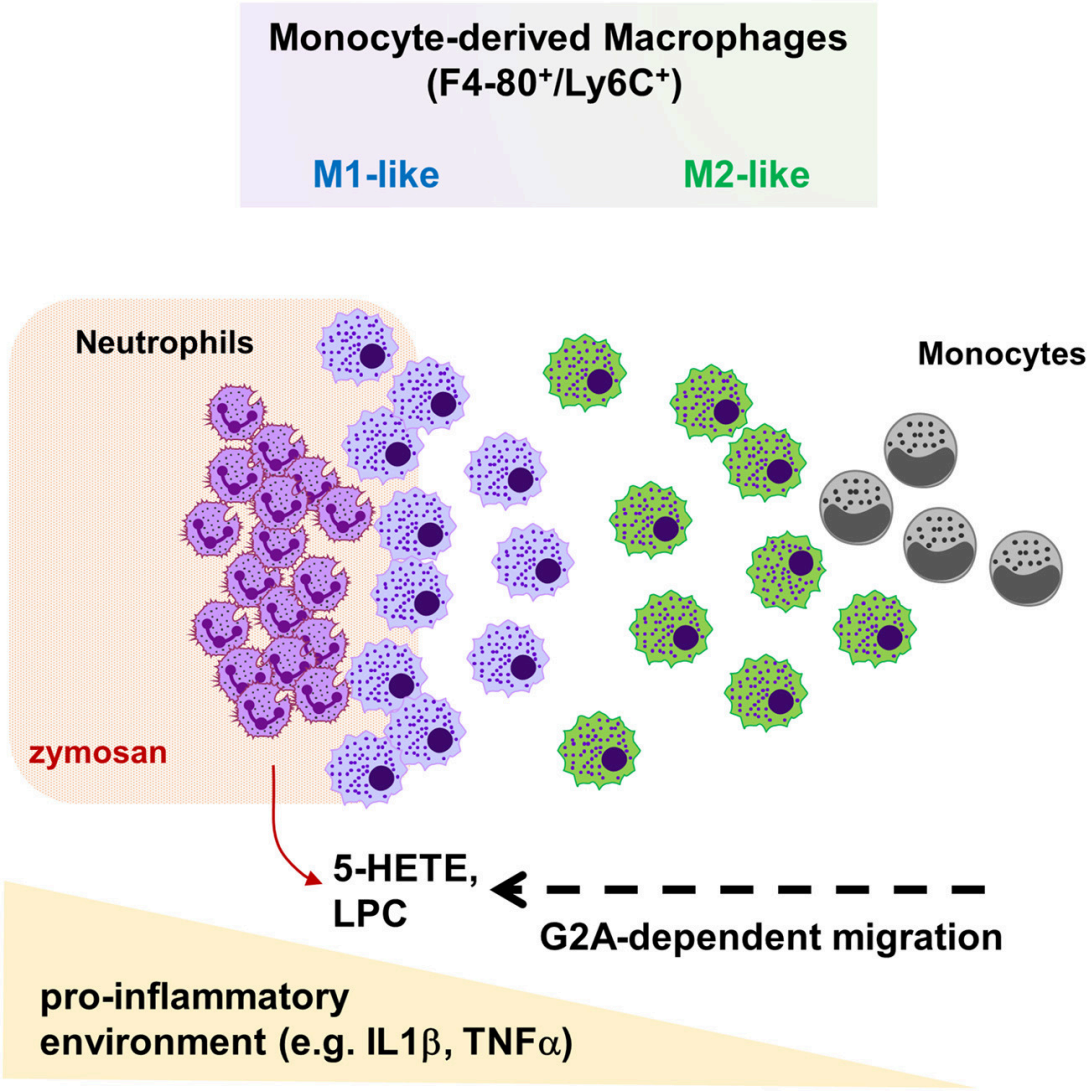
Schematic overview on G2A function during zymosan-induced paw inflammation. Inflammatory monocytes extravasate, differentiate to macrophages and then migrate in dependence of G2A and chemoattractants, such as LPCs or 5-HETE, through the tissue toward the site of inflammation. Thereby, G2A indirectly promotes polarization toward inflammatory M1 macrophages by localizing macrophages in the pro-inflammatory microenvironment at the center of inflammation.

The release of proinflammatory mediators during inflammation causes the recruitment of blood leukocytes, including monocytes, to the inflamed tissue. Here, we show that G2A-deficiency in mice causes a reduced macrophage-dependent thermal hyperalgesia during acute zymosan-induced paw inflammation. This is accompanied by a decreased migration of monocyte-derived macrophages toward the center of inflammation and a reduced neutrophil clearance. Efficient efferocytosis depends on chemotaxis of phagocytes toward the apoptotic cell, which is mediated by “find-me” signals (7, 8, 11). LPCs as well as 5-HETE are likely to be released by neutrophils located at the site of inflammation and, therefore, are candidates to direct macrophage migration. However, since the observed effects on macrophages concentrate on a small defined area, we cannot rule out that other G2A agonists might be locally increased but are not detected using whole tissue analysis. For example, G2A is slightly sensitive for stimulation by protons (17) and a strong local pH-decrease might allow a short range chemotactic effect through G2A. Since we did not observe a decrease of the total number of monocyte-derived macrophages, the data suggest that G2A is not involved in the plasma extravasation of monocytes, but instead regulates chemotaxis within the inflamed tissue. Since the decreased monocyte-derived macrophage numbers are restricted to a very limited area around the center of inflammation, G2A seems to be responsible only for the final positioning of these macrophages in the proinflammatory area within the inflamed tissue (Figure 7).

Alternatively, G2A activation could induce the polarization macrophages toward proinflammatory phenotypes, which is therefore decreased in G2A deficient mice. Yet, G2A-activation by lactate has been demonstrated to induce polarization of bone marrow-derived macrophages and tumor-associated macrophages toward M2-like phenotypes, whereas expression of proinflammatory markers were not affected (20). In support of this report we also did not observe an effect of G2A activation on the expression of M1 markers. Therefore, G2A seems to affect macrophage polarization toward proinflammatory phenotypes indirectly by positioning them in specific microenvironments—in this case the proinflammatory environment around the site of zymosan injection—where they adapt their phenotype accordingly (1, 5).

The observed reduced pain response in G2A-deficient mice can have several reasons. First it has been shown that G2A activation in peripheral sensory neurons allows sensitization of the ion channel transient receptor potential V1 (TRPV1) by G2A contributing to chemotherapy-induced painful neuropathies (18). However, in naïve mice the low neuronal expression of G2A prevents this receptor from participating in nociceptor sensitization and only chemotherapy-induced upregulation of G2A increases the expression levels sufficiently to allow TRPV1 sensitization (18). Since G2A mRNA expression is not upregulated during zymosan-induced inflammation (Figure S4), the involvement of G2A in nociceptor sensitization is unlikely in this model. On the other hand, also the finding that G2A-deficient mice have reduced numbers of proinflammatory macrophages is in accordance with the reduced hyperalgesic response. Proinflammatory macrophages contribute to the classical symptoms of pain, by producing pronociceptive mediators (i.e., TNFα, IL1β, and PGE_2_), which sensitize peripheral sensory neurons (31–33). However depletion of all monocyte-derived macrophages by clodronate reversed the antinociceptive effect seen in G2A-deficient mice suggesting that the macrophages either suppress the release of pronociceptive mediators in the tissue or increase the release of antinociceptive mediators. Indeed, there is a growing list of antinociceptive mediators, which reduce peripheral neuronal sensitization, including polyunsaturated fatty acids (PUFAs), lipoxygenase, and cycloxygenase-metabolites derived from the ω-3 precursors eicosapentaenoic acid (EPA) and docosahexaenoic acid (DHA)—including the resolvin family, maresin as well as DHA and EPA themselves—as well as also other lipids including the ω-3 lipid leukotrien B4 or the ω-9 fatty acid oleic acid. Also cytokines such as TGFβ and IL10 can reduce nociceptor sensitization (31–33). The absence of differences in the level of proinflammatory cytokines between wild type and G2A-deficient mice might point toward the production of antinociceptive mediators, although it should be noted that only some proinflammatory mediators have been studied and therefore both options (reduced pronociception vs increased antinociception) are equally likely until the functional involvement of specific mediators have been demonstrated.

Surprisingly, depletion of all macrophages by clodronate had no net effect on the nociceptive response. Clodronate depletes both, M1-like macrophages, which release pronociceptive mediators that sensitize nociceptors (i.e., IL1β, TNFα, PGE_2_), and M2-like macrophages, which release mediators (i.e., IL10, TGFβ) that can counteract this sensitization. Since the status of the nociceptor sensitization, and therefore the pain response, depends on the balance of the pro- and antinociceptive mediators released within the inflamed tissue (31, 33), the decreased number of M1-like macrophages in G2A-deficient mice can shift this balance toward antinociceptive mediators and a decreased pain response. However, elimination of both, M1- and M2-like macrophages, might reduce pro- and antinociceptive mediators in equal amounts without affecting the overall balance of pro- and antinociceptive mediators in the tissue. Mediators released by other cells in the inflamed tissue seem to be sufficient to maintain a stable nociceptor sensitization (34).

In summary, the data show that the G2A receptor mediates migration of macrophages along a proinflammatory axis toward sources of LPCs and, possibly, 5-HETE during zymosan-induced inflammation and thereby indirectly affects their polarization causing an antinociceptive effect. Thus, G2A seem to fulfill two opposing roles in macrophage polarization. On one side by promoting M2-like phenotypes through direct G2A activation and on the other side by promoting M1-like macrophage phenotypes by positioning macrophages in a proinflammatory microenvironment.

## Author contributions

KK, SS, NT, and JC did the *in vivo* experiments and performed FACS analyses. KK, MS, and SH performed RT-PCRs. GG and NF provided LC-MS/MS analysis data. AW performed FACS sorting. BB provided reagents. KS, TO, and SP performed MELC analysis. KS, KK, and SP designed experiments and wrote the paper. All authors read and approved the manuscript.

### Conflict of interest statement

The authors declare that the research was conducted in the absence of any commercial or financial relationships that could be construed as a potential conflict of interest.

## Abbreviations

BL: baseline
BMDM: bone marrow-derived macrophages
HETE: hydroxyeicosatetraenoic acid
IL1β: interleukin 1β
LPA: lysophosphatidic acid
LPC: lysophosphatidylcholine
LPS: lipopolysaccharide
MELC: Multi-Epitope-Ligand Cartography
PWL: paw withdrawal latency
TNFα: Tumor Necrosis Factor α.
