# Supplementary material for: The G2A Receptor Controls Polarization of Macrophage by Determining Their Localization Within the Inflamed Tissue
Source: Front Immunol. 2018 Oct 1;9:2261. doi: 10.3389/fimmu.2018.02261 (PMC6174245; doi:10.3389/fimmu.2018.02261)
Supplement: Supplementary file 1 [file Data_Sheet_1.PDF]

Supplemental Experiment Procedures

Liquid chromatography-tandem mass spectrometry (LC-MS/MS)

The LC-MS/MS system consisted of an hybrid triple quadrupole-ion trap mass spectrometer QTrap 5500 (Sciex, Darmstadt, Germany), an Agilent 1260 HPLC binary pump, column oven and degasser (Waldbronn, Germany), and a HTC Pal autosampler (Zwingen, Switzerland). High-purity nitrogen for the mass spectrometer was produced by a NGM 22-LC-MS nitrogen generator (cmc Instruments).

LPAs/LPCs:

Sample extraction was performed with liquid–liquid extraction. Therefore, a tissue homogenate containing 0.02 mg tissue/µl PBS was mixed with 10 µL methanol, 20µl of the internal standard (LPC 13:0, 5 ng/ml or LPA 17:0, 1000 ng/mL) and 800 µL water containing 0.1% hydrochloric acid (37%) / methanol (50:50; v/v) and extracted once with 400 µl chloroform. The organic phase was evaporated at a temperature of 45°C under a gentle stream of nitrogen. The residues were reconstituted with 200 µl of methanol, centrifuged for 3 min at 22,238 g, and the supernatants transferred to glass vials before injection (20 µl) into the liquid chromatography-tandem mass spectrometry (LC-MS/MS) system.

For the chromatographic separation, a Luna C18 (2) Mercury column was used (20 x 2 mm inner diameter, 3 µm particle size, and 100 Å pore size, Phenomenex, Aschaffenburg, Germany) with a same material pre-column. A linear gradient was used at a flow rate of 0.4 ml/min for the separation of the analytes with a total run time of 7 min. Mobile phase A was 50 mM ammonium acetate containing 0.2% formic acid and mobile phase B was acetonitrile/ isopropyl alcohol/ formic acid (50:50:0.2, v/v/v). The gradient started with 60% A and was maintained for 0.5 min reaching 5% within another 0.5 min. These conditions were held for 2.5 min. The mobile phase shifted back to 60% A within 0.5 min, and was held for 3 min to re-equilibrate the column.

Quantification was performed with MultiQuant software version 3.02 (Sciex, Darmstadt, Germany) using the internal standard method. Ratios of analyte peak area and internal standard area (*y*-axis) were plotted against concentration (*x*-axis), and calibration curves were calculated by linear regression with 1/x concentration weighting. The coefficient of correlation was at least 0.99. Variations in accuracy were less than 15 % over the range of calibration.

For LPAs: The mass spectrometer was operated in the negative ion mode with an electrospray voltage of -4500 V at 350°C. Multiple reaction monitoring (MRM) was used for identification and quantification. The mass transitions used for quantitation were *m/z* 409.2→153.0 (declustering potential, DP, -80 V, collision energy, CE, -65 V, LPA 16:0), *m/z* 423.2→269.3 (DP -110 V, CE -40 V, LPA 17:0, internal standard), *m/z* 437.2→153.0 (DP -170 V, CE -45 V, LPA 18:0), *m/z* 435.2→153.0 (DP -160 V, CE -70 V, LPA 18:1), *m/z* 433.2→153.0 (DP -90 V, CE -45 V, LPA 18:2), *m/z* 431.2→153.0 (DP -150 V, CE -65 V, LPA 18:3), *m/z* 466.4→153.0 (DP -125 V, CE -34 V, LPA 20:0) and *m/z* 457.2→153.0 (DP -165 V, CE -45 V, LPA 20:4) all with a dwell time of 50 ms.

For LPCs: The mass spectrometer was operated in the positive ion mode with an electrospray voltage of 3500 V at 350°C. Multiple reaction monitoring (MRM) was used for identification and quantification. The mass transitions used for quantitation were *m/z* 496.2→183.9 (declustering potential, DP, 266 V, collision energy, CE, 37 V, LPC 16:0), *m/z* 454.3→184.1 (DP 236 V, CE 33 V, LPC 13:0, internal standard), *m/z* 524.3→184.0 (DP 281 V, CE 39 V, LPC 18:0) and *m/z* 522.3→184.0 (DP 251 V, CE 37 V, LPC 18:1), all with a dwell time of 50 ms.

HETEs:

5-, 12- and 15-HETE were analyzed using liquid chromatography tandem mass spectrometry (LC-MS/MS). Working solutions with 2500 ng/ml of all analytes were prepared in methanol

containing 0.1 % BHT. The calibration standards (0.25- 2500 ng/ml) were prepared by further dilution of the working standards.

Sample extraction was performed using liquid extraction. Therefore, 200µL tissue homogenate were gently mixed with 20µl of internal standard mixture (5(S)-HETE-d8, 12(S)-HETE-d8 and 15(S)-HETE-d8 50ng/ml) and extracted twice with 600 µl ethyl acetate. The organic phase was removed at 45 °C under a gentle stream of nitrogen. The residues were reconstituted in 50 µL methanol/water/BHT (50:50:0.001, v/v/v), centrifuged for 2 minutes at 20,000 g and then transferred to glass vials (AZ-Analytics GmbH, Langen, Germany) prior to injection into the LC-MS/MS system.

The mass spectrometer was operated in the negative ion mode with an electrospray voltage of -4500 V at 550°C. Multiple reaction monitoring (MRM) was used for identification and quantification. The mass transitions used for quantitation were *m/z* 319.1→115.0 (declustering potential, DP, -110 V, collision energy, CE, -20 V, 5-HETE), *m/z* 327.2→58.9 (DP -140 V, CE -44 V, 5-HETE-d8), 319.1→197,1 (DP, -110 V, CE, -20 V, 12-HETE), *m/z* 327.1→214.1 (DP -130 V, CE -20 V, 12-HETE-d8),

319.1→219.2 (DP, -125 V, CE, -20 V, 15-HETE) and *m/z* 327.2→182.0 (DP -150 V, CE -22 V, 15-HETE-d8) all with a dwell time of 50 ms.

For the chromatographic separation, a Gemini NX C18 column and precolumn same material were used (150 mm × 2 mm I. D., 5µm particle size from Phenomenex, Aschaffenburg, Germany). A linear gradient was employed at a flow rate of 0.5 ml/min and a total run time of 17.5 minutes. Mobile phase A was water/ammonia (100:0.05, v/v) and B acetonitril/ammonia (100:0.05, v/v).The gradient started from 85% A to 10% within 12 min. This was held for 1 min at 10% A. Within 0.5 min the mobile phase shifted back to 85% A and was held for 3.5 min to equilibrate the column for the next sample. The injection volume of samples was 20 µL. Quantification was performed with Analyst Software V 1.6.2 (Sciex, Darmstadt, Germany) employing the internal standard method (isotope- dilution mass spectrometry).

Ratios of analyte peak area and internal standard area (y-axis) were plotted against concentration (x-axis) and calibration curves were calculated by least linear regression with 1/concentration weighting.

HODEs:

Lipid identification and quantification was performed as described previously (Sisignano et al., 2016). Briefly, stock solutions with 2500 ng/ml of the analytes: 9-HODE and 13-HODE and the internal standards: 9-HODE-d4 and 13-HODE-d4 were prepared in methanol. Working standards were obtained by further dilution with a concentration range of 0.025-150 ng/ml for all analytes. Sample pretreatment was performed using liquid–liquid extraction. The combined organic phases were removed at a temperature of 45° C under a gentle stream of nitrogen. The residues were reconstituted with 50 µl of methanol/water/BHT ($50:50:10^{-4}$), v/v/v), centrifuged for 2 min at 10,000xg and then transferred to glass vials prior to injection into the LC-MS/MS system.

Multiple reaction monitoring (MRM) was used for identification and quantification. The mass transitions used for quantitation were m/z 295.3→125.2 (declustering potential, DP, -142 V, collision energy, CE, -32 V, 9-HODE), m/z 299.3→172.2 (DP -154 V, CE -26 V, 9-HODE-d4), m/z 295.3→113.1 (DP -151 V, CE -30 V, 13-HODE) and m/z 299.2→198.2 (DP -170 V, CE -26 V, 13-HODE-d4), all with a dwell time of 20 ms.

For the chromatographic separation of oxylipids, a Gemini NX C18 column and precolumn (150 x 2 mm inner diameter, 5 µm particle size, and 110 Å pore size; Phenomenex, Aschaffenburg, Germany) was used. A linear gradient was used at a flow rate of 0.5 ml/min with a total run time of 17.5 min. Mobile phase A consist of water: ammonia (100:0.05, v/v), and mobile phase B of acetonitrile: ammonia (100:0.05, v/v). The gradient changed from 85% A to 10% within 12 min. These conditions were held for 1 min. Then, the mobile phase shifted back to 85% A within 0.5 min and it was maintained for 4 min to re-equilibrate the column.

A volume of 20 µl of the extracted samples were injected into the LC-MS/MS system. Quantification was performed with Analyst software version 1.6.2 (Sciex) and Multiquant software version 3.0.2 (Sciex) using the internal standard method (isotope-dilution mass spectrometry). Ratios of analyte peak area and internal standard area (y-axis) were plotted against concentration (x-axis), and calibration curves were calculated by least-linear regression with 1/square concentration weighting.

References:

Sisignano, M., Angioni, C., Park, C.-K., Meyer Dos Santos, S., Jordan, H., Kuzikov, M., … Geisslinger, G. (2016). Targeting CYP2J to reduce paclitaxel-induced peripheral neuropathic pain. *Proceedings of the National Academy of Sciences of the United States of America*, *113*(44), 12544–12549. https://doi.org/10.1073/pnas.1613246113

Figure S1

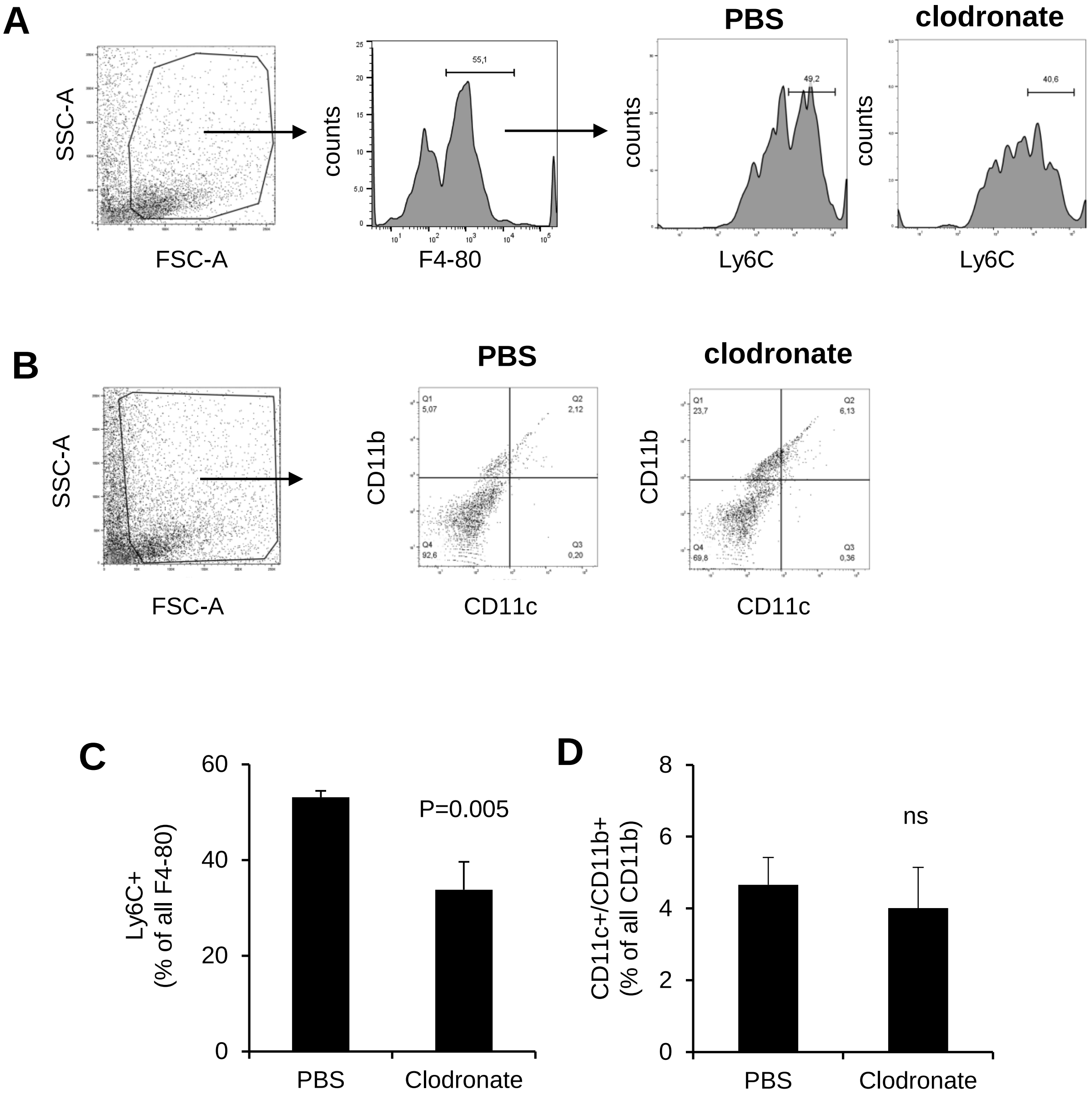

Figure S1. FACS analysis of monocyte-derived macrophages and dendritic cells in inflamed paws.

(A,B) Gating strategies for F4-80$^+$/Ly6C$^+$ monocyte-derived macrophages (panel A) and CD11c$^+$/CD11b$^+$ (panel B) dendritic cells. Cells were isolated from inflamed paws 24 hours after zymosan injection in mice, which received PBS or clodronate-containing liposomes as described in the Methods section.

(C,D) Quantitative analysis of monocyte-derived macrophages (panel C) and dendritic cells (panel D)24 hours after zymosan-injection in mice, which received PBS or clodronate-containing liposomes. Data are presented as mean ± S.E.M.. N=5, Student's T-test, ns =not significant

Figure S2

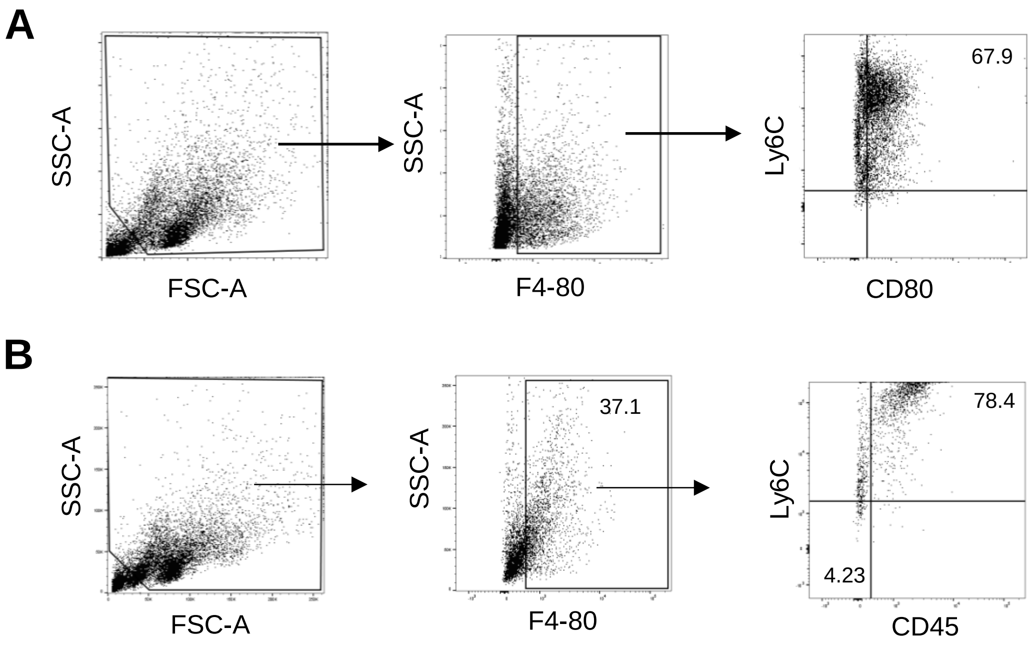

Figure S2. Gating strategy for FACS analysis of macrophages from inflamed paws.
Gating strategies for F4-80$^+$/Ly6C$^+$/CD80$^+$ (panel A) and F4-80$^+$/Ly6C$^+$/CD45$^+$ (panel B) macrophages. Cells were isolated from inflamed paws 24 hours after zymosan injection as described in the Methods section.

Figure S3

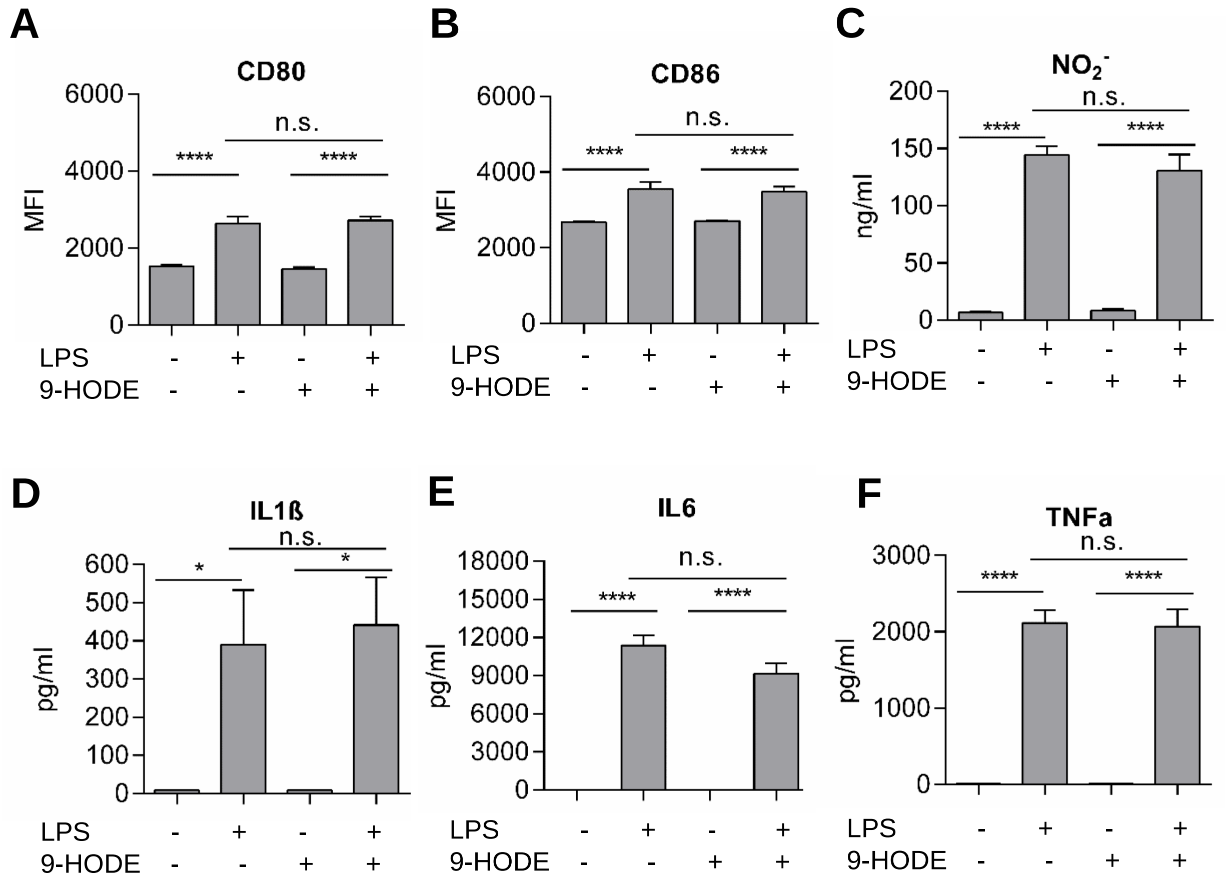

Figure S3. G2A-activation does not alter proinflammatory markers in bone marrow-derived macrophages. (A-F) Marker expression by G2A-deficient bone marrow-derived macrophages after stimulation with 100 ng/ml LPS or 1 µM 9-HODE for 24 h. CD80 and CD86 expression were analyzed by FACS, nitrite using the Griess test and IL1β, IL6 and TNFα by ELISA. Data are shown as mean ±S.E.M. (n=4). One-Way ANOVA with Bonferroni Post-hoc Test *p<0.05, ***p<0.0001, ****p<0.00001.

Figure S4

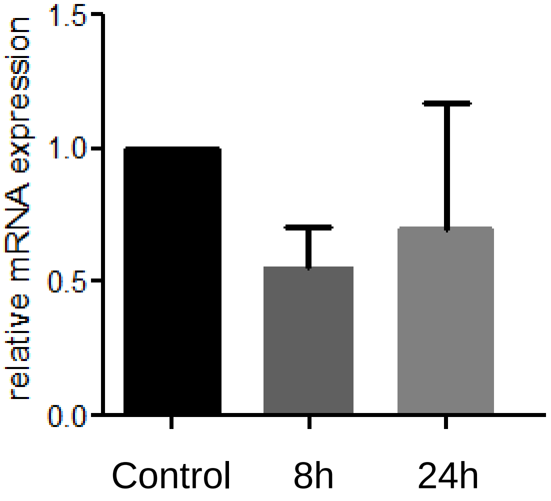

Figure S4: G2A mRNA expression is not upregulated in dorsal root ganglia (DRG) during zymosan-induced inflammation.
Expression analysis of G2A mRNA in L5 DRGs isolated from the ipsilateral side 8 or 24 hours after zymosan-injection. mRNA level were detected by real time RT-PCR. Data are shown as mean ± S.E.M. (n=3). One-Way ANOVA.
